# Addressing the Engagement Blind Spot: 12-Tip Tutorial to Make Reasoning Audible in Health Professions Education

**DOI:** 10.2196/104702

**Published:** 2026-10-20

**Authors:** Inthrani Raja Indran, Dujeepa D Samarasekera

**Affiliations:** 1 Department of Pharmacology, Yong Loo Lin School of Medicine, National University of Singapore, MD3, 16 Medical Drive, Singapore, 117600, Singapore, 65 96832935; 2 Centre for Medical Education, Yong Loo Lin School of Medicine, National University of Singapore, Singapore, Singapore

**Keywords:** student engagement, collaborative learning, clinical reasoning, educational technology, artificial intelligence

## Abstract

Student engagement reforms have increased active learning in higher education. However, visible participation does not always require students to rehearse the spoken, interactive forms of reasoning central to professional practice, such as explaining one’s thinking, responding to challenges, building on others’ ideas, and working through uncertainty in real time. We describe this gap as an engagement blind spot. We define audible reasoning as the deliberate externalization of disciplinary or professional reasoning through spoken interaction so that its assumptions, evidence, uncertainty, and justification can be examined, challenged, revised, and developed collaboratively. It is particularly relevant in health professions education and also applies to laboratory-based and scientific disciplines where students must communicate their reasoning under conditions of uncertainty, scrutiny, and collaboration. Digital, hybrid, asynchronous, and AI-enabled learning environments sharpen this blind spot. Shared documents, online quizzes, discussion boards, and generative AI tools can support participation and access; however, they can also allow students to engage without speaking or to produce polished answers without making the underlying reasoning visible. In this context, educators need to ask not only whether students are engaged but also what behaviors they are rehearsing and whether their reasoning is available for examination and development. This tutorial offers 12 practical, evidence-informed tips developed iteratively from recurring challenges encountered in teaching, curriculum design, and faculty development and refined against selected literature. The tips are organized around 4 clusters covering the design of activities that require audible reasoning, the elicitation of reasoning in practice, the development of reasoning through listening and response, and the preservation of audible reasoning in digital and AI-enhanced environments. Across these 4 clusters, we suggest small but intentional shifts that help educators move from silent, constructive tasks toward audible, dialogic, and professionally relevant participation. These include designing tasks around uncertainty, creating low-stakes opportunities for spoken reasoning, using digital tools to surface and organize contributions for further discussion, structuring active listening and peer feedback, reviewing group dialogue formatively, and using conversational AI as a rehearsal partner rather than an authority. These strategies require attention to psychological safety, cultural responsiveness, accessibility, equity, privacy, data governance, and ongoing monitoring. Audible reasoning is not synonymous with speaking more, volunteering more frequently, or sounding fluent. Designing for audible reasoning is not simply an engagement strategy. It is a way of helping students practice the communicative and collaborative behaviors needed to explain, defend, question, and revise human judgment in clinical, scientific, and other professional contexts.

## Introduction

Over the past two decades, active learning reforms have moved many higher education classrooms away from passive listening and toward more participatory formats, such as in-class polling and online quizzes. Yet a blind spot remains. Many implementations have been designed primarily to deepen individual learning, but they do not consistently require students to rehearse reasoning aloud, although this is central to professional practice. Such rehearsal includes explaining one’s thinking, responding to challenge, building on others’ ideas, and working through uncertainty in real time. We refer to this practice as audible reasoning.

This argument is not intended to dismiss the many dialogic pedagogies already used in health professions education. Case-based learning, team-based learning, problem-based learning, clinical case discussions, bedside teaching, ward rounds, and simulation all create valuable opportunities for interaction and shared sense-making [1]. Yet the presence of such pedagogies does not guarantee that spoken reasoning is consistently rehearsed, made audible, or critically interrogated. Learners may still divide labor, defer to more confident peers, rely heavily on written or AI-generated outputs, or move too quickly toward consensus without articulating the assumptions and judgments shaping their conclusions. The issue, then, is not whether dialogue exists at all, but whether educational designs consistently require learners to think aloud in ways that make their reasoning open to challenge, refinement, and feedback.

This distinction carries particular weight in health professions education, where clinical reasoning is a core capability, although its expression differs across professions and settings [1]. The distinction between participation and reasoning has become more consequential for 2 reasons. First, digital, hybrid, and asynchronous formats have reduced the need for students to speak, allowing them to participate without having to make their reasoning audible. Second, generative AI can now produce polished summaries, explanations, and arguments on demand. A completed answer may therefore provide limited evidence of the reasoning through which it was produced. In technology-enhanced professional environments, information may be readily available. However, learners must still interpret its meaning, define the problem, identify assumptions, determine what matters in context, and justify their position when questioned. AI may help generate or compare possible solutions, but students must still learn not to outsource problem definition itself. This matters not only for professional preparation but also for learning itself.

From a sociocultural perspective, language is not merely a vehicle for expressing thought, but a means through which thought is formed [2]. This tutorial builds on established work in dialogic pedagogy and exploratory talk [3], adapting these principles to higher education, particularly health professions education, including assessment and digital and AI-mediated settings. The ICAP framework distinguishes Interactive, Constructive, Active, and Passive modes of engagement [4]. Constructive, active, and passive activities may occur silently and individually, whereas interactive engagement is inherently dialogic. Many classrooms now move students beyond passive learning but still stop short of requiring them to explain, test, and revise their reasoning with others. Likewise, the Making Thinking Visible movement has helped educators externalize student thinking [5]. However, in higher education, this often takes the form of written artifacts such as reflections, essays, or discussion posts. What may still be missing is deliberate design for the spoken, interactive work of making reasoning available for examination and development.

These behaviors must also be understood in a cultural context. Norms around speaking, questioning, and challenging views vary across educational cultures [6]. In hierarchical settings, restraint may reflect respect rather than disengagement. Designing for audible reasoning should not impose a single dominant style of classroom talk. Instead, it should create predictable, psychologically safe, culturally responsive, and accessible opportunities for learners to practice critical inquiry, professional communication, and reasoned judgment across diverse contexts.

We define audible reasoning as the deliberate externalization of disciplinary or professional reasoning through spoken interaction so that its assumptions, evidence, uncertainty, and justification can be examined, challenged, revised, and developed collaboratively. Audible reasoning is not synonymous with speaking more, volunteering more frequently, or sounding fluent. It draws on related traditions such as making thinking visible and exploratory talk but focuses specifically on designing learning environments in which the grounds for professional judgment become available to others for collective scrutiny and development. Although the construct centers on spoken interaction, this tutorial also considers selected text-based and AI-mediated activities where they preserve or rehearse the same dialogic practices. The question for educators is therefore not only, “Are my students engaged?” but also, “What behaviors are my students rehearsing in my classroom?”

This tutorial presents 12 practical, evidence-informed tips organized into 4 clusters that move from establishing the conditions for audible reasoning to eliciting reasoning in practice, building on the reasoning of others, and sustaining these practices in digital and AI-enabled settings. This tutorial aims to help health professions educators recognize where reasoning remains unarticulated or insufficiently examined and to redesign teaching, feedback, and assessment so that professional reasoning can be heard, challenged, and refined. Textbox 1 lists the learning objectives.

Textbox 1. **Learning objectives.**

1. Explain why visible participation does not necessarily indicate that students are articulating or developing their reasoning.
2. Redesign selected silent or individual learning activities as collaborative tasks that require students to verbalize, examine, and refine their reasoning.
3. Apply strategies that promote psychological safety, reduce anxiety, and support equitable participation in spoken reasoning.
4. Use generative AI for low-stakes conversational practice under educator oversight, rather than as an authoritative source.

## Methods

The 12 tips presented in this tutorial were developed iteratively from recurring challenges encountered in the authors’ teaching, curriculum design, and faculty development work. They were refined against the selected literature cited throughout. The clusters follow the sequence of educational design decisions educators make, beginning with designing for audible reasoning, then eliciting audible reasoning in practice, then developing reasoning through listening and response, and finally sustaining audible reasoning in digital and AI-enhanced environments. The tips are evidence-informed rather than the product of a systematic review or formal consensus process. Table 1 summarizes the 4 guiding clusters and the 12 associated tips.

**Table 1.** Overview of the 4 clusters for making reasoning audible.

Cluster	Illustrative shifts
Design for audible reasoning	Establish expectations and design tasks that require learners to explain, question, justify, and revise reasoning.
Eliciting audible reasoning in practice	Use facilitation strategies in teaching and workplace learning to elicit learners’ reasoning rather than accepting brief or polished answers.
Develop reasoning through listening and response	Structure opportunities for learners to listen actively, question assumptions, build on others’ ideas, and give feedback that advances collective reasoning.
Sustain audible reasoning in digital and AI-enhanced environments	Design digital and AI-mediated activities that preserve dialogue, require uptake of others’ contributions, and use technology to support rehearsal and reflection rather than replace reasoning.

## Results

### Cluster 1: Design for Audible Reasoning

This cluster addresses the first question: Have I designed for audible reasoning by making the expected behaviors explicit and creating tasks that require students to explain, question, justify, and revise ideas with others?

#### Tip 1: Do My Teaching Strategies Require Students to Reason With One Another?

A useful first step is to determine whether a learning activity requires learners to reason with one another, that is, interactive engagement, rather than constructing responses individually, that is, constructive engagement. Many reforms stall at the constructive level, where students may be working hard writing reflections, building slides, solving equations, or preparing answers, but they do so silently or through a division of labor. These activities may deepen individual learning, but they do not necessarily require students to cocreate, defend, or revise ideas with peers. This aligns with research on dialogic teaching and Thinking Together, which suggests that productive collaboration depends on designing tasks in which talk is necessary rather than merely incidental [3].

While critics correctly note that internal cognitive engagement can be difficult to assess simply by observing silent students [7], interactive engagement is different because it is inherently social and observable. A silent classroom may contain effortful learning, but it is not yet rehearsing audible, collaborative reasoning. Educators can use the ICAP framework [4] to audit session plans by asking, “Must learners co-generate, explain, defend, or revise ideas with others to complete this task, or could they complete it independently or by dividing the work?” Identifying where reasoning remains private is the first step toward designing for audible interaction.

The distinction is not merely theoretical. In a quasi-experimental study, Wiggins et al [8] compared constructive and interactive versions of repeated classroom activities on topics such as protein translation and gene regulation in 2 back-to-back introductory biology sections of approximately 350 students each. The activities involved small-group work and addressed the same content. However, the interactive versions incorporated structured peer teaching and synthesis, requiring learners to exchange and integrate information, whereas the constructive versions did not require substantive interaction. The interactive condition produced a modest but statistically detectable improvement in learning. The authors cautioned that the gain was modest and that studies without repeated measures would likely be underpowered to detect it. The findings do not suggest that every spoken activity is superior to constructive individual work. Rather, they illustrate why the quality and necessity of interaction matter when the intended outcome includes collaborative reasoning.

**Barrier and Solution:** Educators may feel they lack the time to audit entire curricula or create space for interactive dialogue within already crowded sessions. Start small by reviewing 1 or 2 core sessions per module and identifying an activity that students can currently complete independently or by dividing the work without engaging with one another’s reasoning. Rather than adding a new activity, redesign or replace this task so that successful completion requires students to reason collaboratively, such as comparing interpretations, explaining the grounds for a decision, responding to an alternative view, or revising a position after discussion.

#### Tip 2: Establish Audible Reasoning as an Explicit Course Expectation

Professional communication behaviors are frequently listed as broad graduate attributes, but they often remain less visible in the daily fabric of the classroom. Educators can establish audible reasoning as an explicit expectation from the outset. This means telling students not only that they are expected to participate but also what kind of participation matters. Students should be expected to explain the grounds for a judgment, respond to challenges or alternative perspectives, question ideas respectfully, and revise their positions in light of better evidence or arguments.

These expectations should be communicated clearly using context-specific language, together with a brief explanation of why interactive methods are being used, as understanding the rationale can reduce student resistance to active learning [9]. Educators should make explicit that the emphasis is on explaining the basis for a judgment and engaging with alternative perspectives, rather than simply speaking more or producing polished answers. The form this takes will vary by context. Students might be asked to critique the methods and limitations of research papers, compare possible management approaches, or justify why they favor one interpretation over another.

What distinguishes audible reasoning from generic participation is that the expectation targets the quality of reasoning rather than airtime. The operative instruction is not simply to contribute but to make the reasoning or grounds for judgment available for others to examine, question, and build on. Students should also understand that this does not require them to speak frequently, respond immediately, or display polished fluency. In hierarchical or culturally reticent settings, designing for audible reasoning may involve predictable prompts, individual preparation, paired rehearsal, and structured roles before students contribute in larger groups.

Where audible reasoning contributes to assessment, educators should publish criteria that focus on the use of evidence, the clarity of justification, responsiveness to other perspectives, the acknowledgment of uncertainty, and the revision of judgment. Speaking frequency, confidence, or verbal fluency should not be used as proxies for *reasoning quality*. Self-reported confidence has been shown to correlate only weakly with objectively assessed competence among health professions students [10]. Students should therefore become familiar with these expectations through repeated low-stakes practice before applying them in summative assessment. This explicitness signals to students that speaking is not merely optional participation but a required course competency (Textbox 2).

Textbox 2. **Tip 2—In practice.**

At the first session, name the expectation explicitly and explain its rationale. The statement can be included in the syllabus and repeated before early interactive activities so that students encounter it as a consistent course norm rather than a one-off instruction: We are not aiming for perfect or polished answers, or for everyone to speak frequently. What matters is that when you contribute, you make your reasoning audible and explain how you arrived at your judgment, what evidence you are drawing on, where uncertainty remains, and what might change your view. You will often have time to think or rehearse with a partner first. Confidence or verbal fluency is not a substitute for the quality of your reasoning.

**Barrier and Solution:** Students may view course expectations as mere suggestions if they are not reflected in the design of learning activities or assessments. Address this by linking audible reasoning to relevant professional standards, framing dialogue as essential career preparation, and intentionally building in recurring opportunities to practice these behaviors during class. Even when not graded directly, consistent use of prompts, structured roles, and formative feedback signals that audible reasoning is part of how learning occurs. Where audible reasoning contributes to assessment, provide explicit criteria and opportunities for low-stakes rehearsal in advance.

#### Tip 3: Design Tasks Anchored in Uncertainty, Not Just Factual Recall

Disciplinary dialogue can feel superficial when students are asked to discuss facts. When the answer is clear, students may default to cumulative talk, in which they agree uncritically with one another, or to disputational talk, in which the emphasis shifts from collective understanding to defending individual positions [11]. To foster more genuine dialogue, educators can design prompts or scenarios that encourage exploratory talk by presenting situations in which facts alone are insufficient to determine the best course of action. For instance, a case in which 2 reasonable management options carry different risks or a dataset that 2 valid methods would interpret differently gives students something they must reason through aloud rather than simply recall. When the pathway to a solution is unclear, students are prompted to reason aloud, examine alternatives, and navigate ambiguity together. In this form of exploratory talk [11], students collaboratively examine, challenge, and constructively build on one another’s ideas. In controlled and replication studies, groups taught the ground rules of exploratory talk outperformed untaught control groups on problem-solving tasks, and these gains transferred to individual reasoning tests rather than remaining confined to the group [12].

However, designing for uncertainty requires students to feel safe enough to be uncertain. Educators also need to resist the urge to resolve discomfort too quickly because students need time to think, sit with ambiguity, and test partially formed ideas before a polished answer is supplied [13]. Students often default to silence because they fear being wrong in front of peers, particularly when psychological safety has not been deliberately cultivated. Educators can reduce this fear by reassuring students that their reasoning need not be complete or correct before it is shared and examined.

Educators can also model the provisional reasoning they hope students will adopt. When teachers present only polished, linear solutions, students may infer that competent reasoning is immediate and certain. Thinking aloud through a novel problem, naming assumptions, identifying what further information is needed, demonstrating how to seek that information, or revising a position in response to a counterpoint can normalize uncertainty and support psychological safety [14].

This approach aligns with the modeling component of cognitive apprenticeship as applied to clinical reasoning education, in which educators deliberately externalize otherwise tacit reasoning processes, including points at which their reasoning remains uncertain or incomplete [15]. Educators can make use of both planned and spontaneous opportunities. When faced with a question they cannot answer immediately, they can acknowledge the uncertainty, explain how they would investigate it, and return later to confirm or revise their position. They can also design incomplete cases, competing interpretations, or deliberately ambiguous steps that allow students to hear what information-seeking, self-correction, and revision sound like in their discipline.

As exploring uncertainty takes time, educators should structure activities to allow meaningful exploration while preserving a clear path to closure. A guiding provisional synthesis, supported by appropriate resources or next steps, can help students distinguish what is well supported, what remains uncertain, and how outstanding questions might be pursued, reducing the risk that misconceptions persist. The aim is not uncertainty for its own sake but to help students make reasoned judgments based on the available evidence while recognizing what remains uncertain.

**Barrier and Solution:** Students accustomed to clear right-or-wrong answers may initially feel frustrated by ambiguous tasks, and both students and educators may fear losing credibility when they appear uncertain in public. Address this by framing “navigating uncertainty” as the exercise’s primary objective before it begins and by treating errors as normal moments in the reasoning process. When a student offers an incomplete or incorrect response, acknowledge the contribution, identify what is useful in it, and model how to refine the idea rather than simply marking it wrong. This helps students see that professional reasoning is not error-free performance but the ability to recognize, correct, and build on emerging understanding.

### Cluster 2: Eliciting Reasoning Aloud

This cluster addresses the second question: Am I eliciting students’ reasoning in the classroom by drawing out their thinking rather than simply accepting polished answers?

#### Tip 4: Step Away From the Podium and Move Into the Room to Elicit Reasoning Across the Class

Large lectures can easily reinforce a content delivery dynamic in which the educator speaks from the front, and students respond only when directly invited. To make thinking audible at scale, educators can shift from delivering content to facilitating reasoning by redistributing attention away from the educator and across the class. Where the physical setting permits, this may begin by stepping away from the podium and moving into the room. When a student answers a question, resist the urge to validate it immediately. Instead, invite responses from elsewhere in the room: “Who interprets this data differently?” or “How does your proposed mechanism build on what your classmate just said?” The aim is to make one student’s answer available for others to examine, extend, or challenge, rather than allowing it to return immediately to the educator for evaluation. In this way, the educator’s movement helps redistribute attention across the room and prompts students to listen actively and reason collaboratively, even in a large lecture hall.

Digital tools can complement this process by making students’ contributions available for comparison, organization, and further discussion, rather than merely collecting answers. One example is Talkwall [16], a microblogging tool through which students submit brief contributions individually or in groups to a shared feed. Selected contributions can then be moved to a shared wall and spatially organized, helping recurring themes, contrasting perspectives, and relationships among ideas become visible. In teaching health professions, a comparable digital wall might be used to organize possible diagnoses, differentials, competing interpretations of a dataset, or arguments for different management options. Figure 1 illustrates one way a shared digital wall can surface different responses to a question and then return them to spoken, collaborative examination.

**Figure 1.**
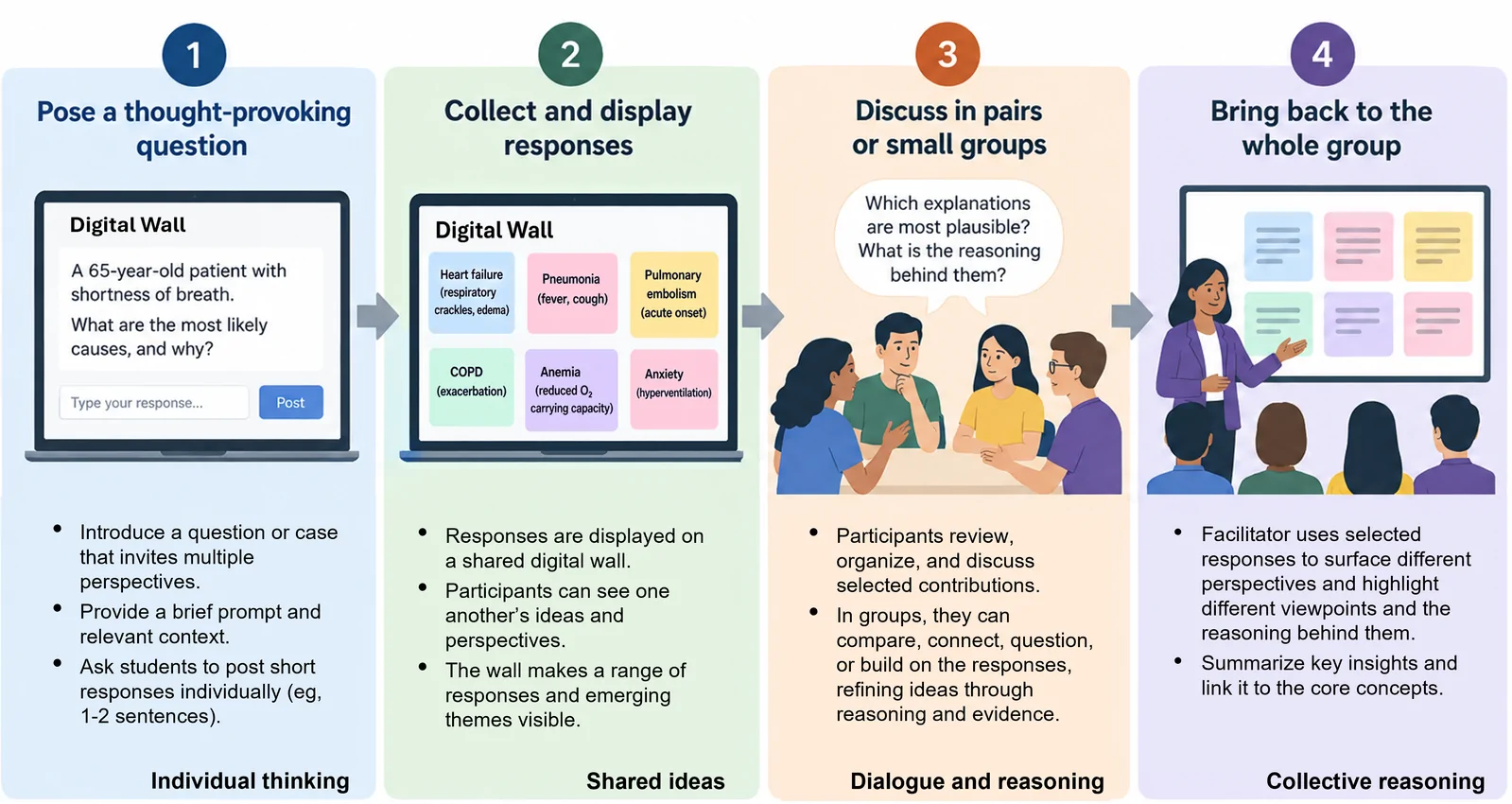
Using digital walls to seed spoken dialogue. Digital walls can make students’ initial ideas visible and provide a shared starting point for discussion when educators structure how students engage with and build on one another’s contributions. COPD: chronic obstructive pulmonary disease; O_2_: oxygen.

Live polling followed by paired explanations and repolling can similarly show whether students reconsider their positions after discussion. At the same time, shared annotation tools can support the comparison of interpretations of a clinical image, research figure, or case. In each instance, digital contributions should seed and structure spoken dialogue rather than replace it [3].

The same principle applies to synchronous online teaching, where a videoconference space replaces the physical room. Here, an educator cannot move through the room but can achieve a similar redistribution of attention by naming students to extend or challenge a peer’s point, routing a contribution to a breakout group for discussion before it returns to the whole class, or using the chat as a staging space from which spoken responses are then invited. The aim is unchanged. One student’s reasoning should remain open for others to examine rather than be closed by the educator’s evaluation.

**Barrier and Solution:** Students often find it intimidating to speak across a large lecture hall. This can be mitigated using a brief think-pair-share activity [17], allowing students to rehearse their ideas with a peer before sharing them with the wider room. When movement around the room is constrained by room design, class size, or accessibility considerations, educators can use roaming microphones, wireless slide clickers that preserve freedom of movement, student facilitators, paired spokespersons, or selected digital responses as prompts for discussion. The pairing step is what makes this approach workable at scale: experimental work shows that frequent, warmly framed invitations to speak can increase the number of students who participate voluntarily, with participation growing over the course of a semester [18]. It is worth being candid, however, that the “share” phase is not risk-free. Some students may still feel anxious when asked to speak in front of the class, especially if they have not volunteered. Think-pair-share should therefore be treated as a scaffold that lowers the threshold for participation, not a guarantee to remove anxiety. We return to this issue later when considering the adverse effects of audible reasoning.

#### Tip 5: Transform Practical and Laboratory Procedures Into Troubleshooting Dialogues

Practical and laboratory sessions are often cognitively demanding, leading students to follow protocols procedurally rather than reason aloud. Collaborative troubleshooting is frequently absent, although recent work shows that structured dialogue in laboratories can enhance conceptual understanding and engagement [19]. Introduce structured “stop-and-talk” checkpoints at critical junctures or at the end of the class to review the rationale for the protocol steps or investigate anomalous data. For instance, before leaving the class, require students to discuss the session’s core rationale with their peers and verbally demonstrate their understanding to the facilitator. If a group cannot reach consensus, they can be asked to articulate their differing interpretations openly so that uncertainty becomes a resource for discussion rather than something to hide. This shifts the laboratory from a space of procedural completion to one in which students rehearse real-time reasoning, troubleshooting, and collaborative problem-solving (Textbox 3).

Textbox 3. **Tip 5—In practice.**

Insert 1 “stop-and-talk” checkpoint before students leave. Ask each pair or group to answer 3 questions aloud to the facilitator:

“What are you doing at this step, why are you doing it, and how would you interpret a result that differs from what you expected?”

If students in the group disagree about the interpretation, ask them to articulate both explanations and identify what additional information, observation, or test would help distinguish between them.

Used this way, the checkpoint can replace a redundant procedural step rather than simply adding another activity to the session, while allowing the facilitator to probe understanding that may not be apparent from a written report.

**Barrier and Solution:** Faculty may find it difficult to add discussion checkpoints to already tight, time-pressured laboratory schedules. One way to address this is to replace a redundant procedural step with a single high-impact verbal checkpoint, so that dialogue replaces rather than adds to students’ cognitive load. In its simplest form, this checkpoint functions as a brief, low-stakes teaching strategy: students explain what they are doing, why they are doing it, and how they would interpret an unexpected result.

Where appropriate, such checkpoints could also be developed into a partial alternative to the traditional written laboratory report. In this more formal assessment role, the oral format offers an important advantage: the examiner can probe students’ responses, which may reveal dimensions of understanding that a static written report does not capture. Oral assessment does, however, carry known risks of reliability and bias, particularly under high-stakes or poorly structured conditions [20]. If verbal checkpoints are used for grading, they should be structured with common prompts and explicit criteria and include appropriate examiner calibration and student accommodations to ensure fairness and interexaminer consistency.

#### Tip 6: Move Students From Scripted Delivery Toward Conversational Explanation

When students deliver presentations, they may rely heavily on scripted notes or read directly from text-heavy slides, stifling spontaneous, dialogic reasoning. To develop the habits of an agile professional, educators should help presenters move from memorized scripts toward explaining ideas conversationally, requiring them to internalize core concepts and to use cue cards strictly as brief prompts. To ensure students prioritize this practice over mere content delivery, educators should include clarity and coherence of explanation, responsiveness to questions, adherence to time limits, appropriate pacing, audience engagement, and equitable sharing of questions and answers in the grading rubric [21]. These communication behaviors should be assessed separately from the quality and justification of the underlying reasoning, as outlined in tip 2. Sharing this rationale up front helps students understand that time management and collective team defense are core professional skills, preventing a dynamic in which one confident student dominates the dialogue.

Expecting the team to pause and field questions organically transforms a static delivery into a genuinely interactive defense. This kind of spoken presentation is not merely a classroom exercise but a recognized vehicle for clinical learning.

In a recent mixed methods study, medical students described preparing and delivering oral case presentations at academic conferences as strengthening their later clinical training and professional development, which suggests that practicing conversational, defensible explanation can carry value well beyond the immediate task (Textbox 4) [22].

Textbox 4. **Tip 6—In practice.**

Treat the presentation as a conversation, not a script. Pause at a key decision point and probe the reasoning directly:

“You’ve told me the plan. Now, tell me why. What in the patient’s history or data pushed you towards this and away from the next most likely alternative? What are you still uncertain about, and what finding or piece of information would change your mind?”

Clinical reasoning frameworks can provide useful scaffolds by helping students organize information and develop a systematic approach to case presentation. As students progress, these frameworks may become more integrated into how they synthesize the case, rather than being followed as a rigid sequence. At the same time, educators should continue to probe the reasoning underlying key decisions so that students develop both an organized approach to presenting the case and the ability to explain and defend their clinical judgment.

**Barrier and Solution:** Removing scripts can significantly increase presenter anxiety among some students, particularly for nonnative speakers of the language of instruction. Mitigate this by affording reasonable flexibility where needed, such as allowing slightly more detailed prompts or additional structure, while maintaining the broader goal of developing more conversational explanation. Scaffold the experience throughout the course. Early on, students may benefit from structured formats, cue cards, or informal seated group updates to help organize their presentation, but these scaffolds should not postpone conversational probing of their reasoning. From the outset, educators can ask students to explain why they favored a particular interpretation, what evidence supports it, what uncertainty remains, or what information would change their position. The nature and degree of scaffolding can be tailored to the individual student. As students progress, these supports can become more flexibly incorporated into how they synthesize and present a case. The key question is what behavior the student is rehearsing. Rather than repeatedly practicing scripted delivery, students can be gradually supported to explain, adapt, and respond in real time, behaviors they will need in professional settings.

### Cluster 3: Building on the Reasoning of Others

This cluster addresses the third question: Are students building on one another, listening and responding rather than delivering parallel monologues?

#### Tip 7: Cultivate Active Listening That Seeks to Understand, Not Just Respond

Student presentations can easily leave the audience passive, with students tuning out until it is their own turn to speak. Educators can rebalance this dynamic by treating the audience as active participants in a professional exchange rather than as observers. As established in cluster 1, audible reasoning needs to be explicitly expected. This tip extends that expectation to the listener: reasoning only becomes dialogic when peers listen closely enough to understand, question, and build on what has been said.

Active listening is too often assumed to be innate or passive, when it is better understood as an active, teachable professional practice [23]. It draws on the same capacities whether a clinician is listening to a patient, a trainee to a senior, or a student to a peer presenting. In medicine, listening supports diagnosis and the therapeutic relationship [24]. The classroom is therefore an important rehearsal space for a competency that professional practice constantly demands.

Begin by making attentive listening visible. Establish explicit rules of engagement for peer presentations, including specific periods when students close their laptops and silence their devices to direct their attention toward the speaker. Students who require devices for accessibility should continue to be accommodated. This signals that giving a colleague undivided attention is professional behavior, not merely a courtesy. However, attention alone is not enough. The aim is to listen to understand before replying. This means following a peer’s reasoning, noticing the inferences one is already forming, and holding one’s assumptions in check rather than projecting one’s own meaning onto what was said. Only then can a listener respond with curiosity rather than waiting to rebut, correct, or display knowledge. Active listening can also be designed into the task itself by making each student’s contribution dependent on understanding what has already been said.

This matters because building on another person’s reasoning depends on first understanding it. A question asked to clarify, probe assumptions, offer another perspective, or identify a gap can extend the presenter’s thinking. In contrast, a question asked only to challenge or showcase knowledge may close dialogue. Inattentive listening is not merely a missed opportunity; it can derail discussion or lead the group away from the reasoning that needs attention. Educators can make this expectation concrete by assigning rotating listening roles, such as clarifier, evidence seeker, assumption checker, or connector. Each role directs attention to a different aspect of the presenter’s reasoning and gives the student a defined contribution to make after the presentation. Alternatively, each audience member could prepare 1 question that clarifies meaning, 1 comment that connects the talk to an earlier idea, and 1 point that respectfully tests an assumption. These moves align with the response practices through which listening becomes visible in group talk, including revoicing, integrating, interpreting, and critically extending what others have said [25]. In doing so, the audience shifts from passive listeners to active participants who practice the professional behaviors of attending, interpreting, questioning, and building on others’ reasoning (Textbox 5).

Textbox 5. **Tip 7—In practice.**

In a preclinical class, the educator can portray a patient with a predetermined history and initially present with only a single symptom, such as headache. Further information is revealed only in response to appropriate questions. Students take turns assuming the physician’s role and ask 1 history-taking question at a time as a microphone is passed around the room. Each student must listen to what has already been asked and elicited, avoid unnecessary repetition, and decide what still needs to be explored. At the end, another student summarizes the history, identifies salient positive and negative findings, and notes any important gaps. The activity makes listening observable through the relevance of each successive question and the accuracy and completeness of the final synthesis.

**Barrier and Solution:** Students may request access to their laptops to take notes or access course materials. In interactive sessions where students are expected to listen and respond, explain that limiting device use during these periods supports real-time listening, understanding, and engagement rather than simply receiving information. Provide paper for brief notes if needed or ask presenters to circulate a 1-page summary before the session so that the audience can focus less on transcribing content and more on the reasoning presented. Make reasonable exceptions when devices are required for accessibility or other learning needs.

#### Tip 8: Break the “Parallel Monologue” in Digital Forums

Asynchronous discussion forums frequently devolve into parallel monologues, with students posting isolated thoughts to meet a quota rather than engaging in genuine exchange [26]. Just as active listening in the room turns an audience into participants, the same disposition is needed online: reading peers’ contributions to genuinely understand them, weighing those views with curiosity and openness, and offering one’s own in response rather than in isolation. Sustained critical inquiry in text-based forums depends on precisely this kind of engagement, in which participants connect and build on one another’s contributions rather than posting in parallel [27]. To foster this, educators might reconsider the common practice of asking students to post original responses to a static prompt.

Instead, forums can be designed around synthesis and critical review, requiring students not only to articulate their own perspectives but also to engage directly with the substance of their peers’ contributions, creating a more interactive and evolving conversation. Educators can also bring selected forum posts into class, using contrasting interpretations or unresolved questions to prompt discussion of assumptions, evidence, and possible revisions. Discussion forums are a setting in which the engagement blind spot is especially apparent because text-based tools make it structurally easy to contribute without reasoning with anyone. Therefore, the same design intent that animates the spoken classroom, making thinking available for others to examine, question, and build on, must be carried deliberately into asynchronous space rather than assumed to follow from participation alone (Textbox 6).

Textbox 6. **Tip 8—In practice.**

Replace a generic “post and reply” instruction with one that requires students to engage directly with a peer’s reasoning:

“Share your response to the case questions. Then choose 1 peer’s contribution and refer to a specific point they have made. Explain 1 point you agree with and why, identify 1 claim you find uncertain or under-supported, and pose 1 question that could move the reasoning forward.”

Where feasible, bring 2 contrasting discussion threads into the next live session and ask the class to examine where the students’ reasoning diverged and why.

**Barrier and Solution:** In discussion forums, when asked to respond to peers, students may default to a brief, polite agreement before launching into a new, unrelated monologue. Overcome this by providing explicit instructional guidance on how to engage. Prompt them to identify which elements of their peer’s post they agree with, which they find uncertain or challenging, and which require clarification or evidence, and to explain why. Furthermore, explicitly reiterate the ground rules for respectful, nonjudgmental exchange, reminding students that their peers’ perspectives may be shaped by diverse life, educational, and clinical experiences.

#### Tip 9: Scaffold Peer-to-Peer Feedback Using Structured Templates and Feedback Prompts

Effective peer feedback is a professional skill that requires students to identify strengths, recognize areas for improvement, and communicate both respectfully and constructively. In the context of audible reasoning, it also gives students repeated practice in listening to another person’s contribution, interpreting its value, and offering a response that helps the person improve. It can be enhanced through structured scaffolds [28]. Yet many students struggle to do this well because they may lack the vocabulary, confidence, or a sense of psychological safety to express their judgments clearly. Peer feedback can be scaffolded in the earlier years of higher education, particularly through group projects, using structured templates, cue words, sentence stems, and guided prompts that help students focus on specific examples, explain the impact of behavior, and suggest practical next steps. The aim is to move feedback away from vague praise or vague criticism and toward a disciplined form of professional dialogue in which students identify a specific behavior, explain its impact, and suggest how it might be strengthened.

The process can be further strengthened when recipients of the feedback evaluate its quality using a simple matrix that considers whether it was constructive, respectful in tone, and clear and actionable in content. This makes feedback a 2-way learning process in which students practice giving useful feedback while also learning to assess whether the feedback they receive is specific enough to support improvement. In a group setting, each student evaluates the feedback directed to them, followed by a brief group debrief on common strengths and areas for improvement. Faculty may sample the matrices to guide the process rather than grade every exchange. As students progress through the years, these scaffolds can be gradually faded as constructive peer dialogue becomes more fluent and internalized (Textbox 7).

Textbox 7. **Tip 9—In practice.**

Provide students with a format that separates the strength, the issue, and the actionable next step, and require 1 of each:

“One strength you demonstrated was inviting quieter team members into the discussion [specific behavior], which helped the group hear a wider range of perspectives [positive outcome/impact].”

“One area to strengthen is explaining your reasoning more explicitly when you disagree [behavior to improve], as this would make your contribution clearer and easier for others to engage with [desired outcome/impact].”

“A helpful next step might be to state your rationale before offering an alternative view [specific suggestion].”

Then ask the recipient to rate the feedback they received on 3 dimensions. Was it constructive, respectful in tone, and specific enough to act on? Close with a brief group debrief on common patterns. This allows students to rehearse both giving useful feedback and judging the quality of feedback they receive, rather than simply exchanging vague praise or criticism.

**Barrier and Solution:** Students may avoid honest critique for fear of harming relationships, leading to vague or overly softened feedback. This can be addressed by structuring both the giving and receiving of feedback. Requiring 1 strength and 1 area for improvement encourages balanced commentary. In this way, peer feedback becomes a safe and structured practice for developing professional judgment, communication, and reflective capacity.

### Cluster 4: Sustaining Audible Reasoning in an AI-Enhanced Era

This cluster addresses the fourth question: Does the practice hold up in technology- and AI-enabled settings, where reasoning most easily falls silent? Tip 10 focuses on preserving spoken reasoning in technology-mediated participation, tip 11 focuses on reviewing dialogue after it has occurred, and tip 12 focuses on using conversational AI to rehearse future interactions.

#### Tip 10: Set Ground Rules for Spoken Participation in Technology-Mediated Spaces

Technologies that lower the barrier to participation, such as shared documents, discussion boards, and typed chat, can support access and broaden participation. However, they can also make it structurally easy for students to engage without ever speaking. Silent typing during in-person group work or prolonged silence in online sessions is not simply a sign of poor motivation; it is often a predictable outcome of how participation has been designed. Educators can respond by establishing clear structural expectations for spoken interaction. Even where digital tools are used to surface and organize contributions, as discussed in tip 4, educators should decide which stages of the activity require students to negotiate, justify, or synthesize ideas aloud.

For example, in in-person group work, the first 5 minutes of a collaborative task might be designated as entirely “tech-free,” allowing only brief handwritten notes, with appropriate accommodation for students who require devices for accessibility. Students must first brainstorm, negotiate, and justify ideas aloud before shifting to shared documents or AI tools. Once an initial position has been articulated, these technologies can be used to search for evidence, compare alternatives, or develop the group’s synthesis.

In online settings, educators can assign explicit interaction roles to ensure that spoken contributions are distributed rather than left to a few confident voices. The point is not to reject technology but to decide deliberately when it supports reasoning and when it quietly replaces spoken interaction (Textbox 8).

Textbox 8. **Tip 10—In practice.**

Sequence the use of technology into the lesson structure:

Divide a collaborative task into spoken and digital phases and make the sequence explicit to students:

“For the first five minutes, discuss the problem without using digital or AI tools. Brainstorm, compare your views, and explain your reasoning aloud. Then use the shared document or AI tools to search for evidence and compare alternatives. Finally, return to your discussion and explain what your group retained, changed, or rejected, and why.”

Where students require devices for accessibility or other learning needs, appropriate accommodation should be maintained. The purpose is not to exclude technology, but to ensure that it supports the group’s reasoning rather than replacing the opportunity to reason together.

**Barrier and Solution:** Students may cite screen fatigue, privacy concerns, or discomfort with being visibly present online. This can be addressed by using a professional compromise where we allow flexibility during lower-interaction portions of the session, while clearly identifying selected moments where verbal and, where feasible, visible participation is expected. This respects student well-being while still rehearsing the norms of contemporary professional communication.

#### Tip 11: Use Recordings and AI-Assisted Analysis to Reflect on Participation and Dialogue

Once reasoning has been made audible, students can benefit from looking back at how their group dialogue unfolded. Short recordings or transcripts of group discussions can be used as reflective artifacts. With appropriate consent and data safeguards, educators may ask students, or an institutionally approved AI tool, to review a discussion for whether the group demonstrated patterns of collaborative reasoning, such as inviting contributions, clarifying or revoicing ideas, integrating different viewpoints, extending the discussion through questions, and revising positions in response to others [29]. The review can also consider whether these practices were distributed sufficiently to support collective reasoning. These prompts draw on response practices that make listening visible in group talk, from acknowledging and revoicing to integrating and critically extending what others have said [25].

Reviewing group interaction in this way connects directly to the active listening in cluster 3 and lets students see whether they are building on one another’s reasoning or merely taking turns speaking. For example, a group might review a brief excerpt of their interaction transcript to identify 1 instance in which members developed an idea collaboratively, note 1 missed opportunity to clarify or integrate a contribution, and agree on 1 practice to strengthen in its next discussion.

The purpose should be formative rather than punitive. AI may help surface observable patterns in listening and response, but these outputs should be treated as prompts for reflection rather than definitive judgments of competence. Educators should avoid reducing participation to simplistic metrics such as the number of turns, speaking time, or frequency of keywords because these may reward confidence or verbosity rather than meaningful contribution. AI-generated interpretations may also overlook context, tone, nonverbal communication, or the disciplinary significance of a contribution. They should therefore be checked against the recording or transcript and interpreted alongside group self-reflection, peer feedback, and educator judgment.

**Barrier and Solution:** Recording discussions raises legitimate concerns about privacy, consent, and the possibility that students will perform for the recording rather than participate naturally. Address this by clearly explaining the educational purpose, obtaining appropriate consent, minimizing identifiable data, using only institutionally approved tools that comply with appropriate data safeguards, keeping the focus formative, and deleting recordings and transcripts once their educational purpose is served. Do not upload identifiable material to publicly available AI tools. Framing the review as a shared, low-stakes reflection on how the group reasons together, rather than as surveillance of individual performance, may help preserve the psychological safety on which honest participation depends.

#### Tip 12: Use Conversational AI to Expand Opportunities for Rehearsing Audible Reasoning

Generative AI is often discussed as a tool for producing notes, summaries, and written responses, but one promising educational value may lie in creating additional opportunities for spoken rehearsal. In health professions education, and in adjacent scientific or laboratory-based disciplines, students must learn not only to arrive at a plausible answer but also to explain their reasoning clearly, respond to challenges, defend their decisions, and adapt their positions in real time. Conversational AI tools can support this by serving as low-stakes rehearsal partners beyond the limited hours of classroom time. While the previous tip used AI to review reasoning that had already taken place, this tip looks ahead, allowing students to rehearse reasoning before they have to perform it in class, clinical, or professional settings.

Educators may design simple, class-specific conversational AI activities that allow students to rehearse the forms of spoken reasoning and professional dialogue required in their discipline. Students might use these tools to simulate a presentation audience, respond to follow-up questions, defend weak points in an argument, or receive provisional formative feedback on clarity, structure, pacing, coherence, and communicative style. AI-generated questions and feedback may nevertheless be inaccurate, biased, overly confident, or poorly aligned with disciplinary expectations. Students should therefore be advised to verify feedback against appropriate evidence and professional standards and treat it as a prompt for reflection rather than an assessment of competence. In clinical or scientific contexts, such tools might also simulate skeptical peers, patients, examiners, or interdisciplinary colleagues. Used in this way, conversational AI does not replace authentic human dialogue but expands access to structured rehearsal for the audible, interactive skills that professional practice demands.

Early evidence is encouraging but should be read with care. In a mixed methods study, medical students at 3 universities used a conversational AI system as a simulated patient to rehearse demanding encounters, such as breaking bad news and counseling anxious patients. They reported gains in confidence and perceived usefulness [30]. In a study of nurse practitioner students, an AI-driven virtual simulation was used to practice oral case presentations and receive structured formative feedback on how well students communicated their clinical reasoning and demonstrated clinical judgment [31]. In language education, where the evidence base is larger, a controlled mixed methods study found that AI conversation partners can improve speaking performance while reducing speaking anxiety [32]. The important caveat is that most of these outcomes are self-reported confidence or second-language proficiency rather than demonstrated gains in clinical or scientific reasoning. As noted in tip 2, confidence that outpaces competence is itself a recognized hazard. Conversational AI is therefore best positioned as a rehearsal space that lowers the cost of practice, with judgments of competence reserved for human assessment.

**Barrier and Solution:** Students may overrely on AI-generated feedback or treat these exchanges as scripts to memorize rather than opportunities for genuine reasoning. This can be addressed by positioning AI as a rehearsal partner rather than an authority. Ask students to reflect on which suggestions they accepted, rejected, or modified, and why, and to identify feedback that was inaccurate, unsupported, biased, or inconsistent with disciplinary expectations. AI-assisted rehearsal should remain formative and complement, rather than displace, practice with peers, educators, patients, and other professionals. Final judgment should remain grounded in educator feedback, peer discussion, disciplinary expectations, and observed performance with real people. Where exchanges involve identifiable, sensitive, or clinical information, the privacy and data governance safeguards outlined in tip 11, tip 12, and the “Discussion” section should apply.

## Discussion

Compelling students to externalize reasoning carries risks of its own. The most important of these is the psychological safety on which audible reasoning depends. Requiring students to speak when they have not volunteered can raise rather than lower anxiety, driven by a fear of negative evaluation, and this burden is not evenly distributed [33]. Students from underrepresented groups and nonnative speakers of the language of instruction may be disproportionately affected when fluency under pressure becomes the medium of participation. A poorly designed push toward audible reasoning, therefore, risks amplifying inequities and conflating verbal fluency with the quality of reasoning. These risks are mitigable rather than disqualifying.

The safeguards described throughout this tutorial can reduce these risks. These include giving students time to rehearse in pairs before whole-class sharing, using warm, predictable invitations rather than “gotcha” questions, and helping them reformulate a half-formed answer before sharing it with the wider class. When active learning is implemented with attention to inclusion, it can narrow achievement gaps rather than widen them [34]. The design choices advocated throughout this tutorial are therefore not optional refinements. Ground rules, psychological safety, low-stakes rehearsal, and structured participation are the conditions that make audible reasoning safe and equitable.

Where strategies involve recording discussion or routing student reasoning through AI tools, the practical safeguards set out in tips 11 and 12 should be observed. Such recordings and transcripts may contain sensitive student information and should be managed in accordance with relevant institutional policies and legal requirements. A particular concern in any setting is whether a third-party tool can access, store, or reuse student data. Educators should therefore use institutionally approved tools where possible, minimize the amount of identifiable data collected, keep the purpose formative, and delete recordings or transcripts once their educational purpose has been served.

Educators can strengthen implementation by monitoring how these strategies work in their own contexts, including unintended effects such as heightened anxiety or inequitable participation, as well as the quality of reasoning and patterns of participation. They should also examine whether students explain the grounds for their judgments, acknowledge uncertainty, respond to alternative interpretations, revise their positions, and invite or build on others’ contributions. The aim is not simply to make students speak more but to create conditions in which more students can practice reasoning aloud, listen to one another, and build judgment together.

Taken together, the 4 clusters offer educators a compact self-check they can apply to almost any teaching encounter: have I set the conditions for reasoning aloud, am I eliciting that reasoning in the room, are students building on one another’s contributions, and does the practice hold up in technology- and AI-mediated settings? Used deliberately, these moves bridge the gap between individual classroom participation and the collaborative competence that real-world clinical and scientific practice demands. Verbal communication should not be treated as a generic soft skill appended to disciplinary learning but as a fundamental mechanism through which complex thought is formed, tested, and refined. This is what the era of generative AI clarifies rather than diminishes. As machines increasingly assist with producing answers, the distinctive work of education becomes teaching students to voice, defend, and revise human judgment in dialogue with others. Designing for audible reasoning is therefore not simply an engagement strategy, but a way of preparing graduates to participate in the collaborative reasoning that clinical, scientific, and other professional contexts require.

## Abbreviations

GAIDeT: Generative AI Delegation Taxonomy
GenAI: generative AI

## References

[1] Elvén M, Welin E, Wiegleb Edström D (2023). Clinical reasoning curricula in health professions education: a scoping review. J Med Educ Curric Dev.

[2] Vygotsky LS, Cole M, John-Steiner V, Scribner S, Souberman E (1978). Mind in Society: The Development of Higher Psychological Processes.

[3] Mercer N, Hennessy S, Warwick P (2019). Dialogue, thinking together and digital technology in the classroom: some educational implications of a continuing line of inquiry. Int J Educ Res.

[4] Chi MTH, Wylie R (2014). The ICAP framework: linking cognitive engagement to active learning outcomes. Educ Psychol.

[5] Ritchhart R, Church M (2020). The Power of Making Thinking Visible: Practices to Engage and Empower All Learners.

[6] Frambach JM, Driessen EW, Beh P, van der Vleuten CPM (2014). Quiet or questioning? Students’ discussion behaviors in student-centered education across cultures. Studies in Higher Education.

[7] Thurn CM, Edelsbrunner PA, Berkowitz M, Deiglmayr A, Schalk L (2023). Questioning central assumptions of the ICAP framework. NPJ Sci Learn.

[8] Wiggins BL, Eddy SL, Grunspan DZ, Crowe AJ (2017). The ICAP active learning framework predicts the learning gains observed in intensely active classroom experiences. AERA Open.

[9] Andrews M, Prince M, Finelli C, Graham M, Borrego M, Husman J (2022). Explanation and facilitation strategies reduce student resistance to active learning. Coll Teach.

[10] Lai NM, Teng CL (2011). Self-perceived competence correlates poorly with objectively measured competence in evidence based medicine among medical students. BMC Med Educ.

[11] Mercer N (2004). Sociocultural discourse analysis: analysing classroom talk as a social mode of thinking. Journal of Applied Linguistics.

[12] Wegerif R, Mercer N, Dawes L (1999). From social interaction to individual reasoning: an empirical investigation of a possible socio-cultural model of cognitive development. Learn Instr.

[13] Kumagai AK (2022). Discomfort, doubt, and the edge of learning. Acad Med.

[14] Dong C, Altshuler L, Ban N (2025). Psychological safety in health professions education: insights and strategies from a global community of practice. Front Med (Lausanne).

[15] Jagannath AD, Dreicer JJ, Penner JC, Dhaliwal G (2023). The cognitive apprenticeship: advancing reasoning education by thinking aloud. Diagnosis (Berl).

[16] Smørdal O, Rasmussen I, Major L (2021). Supporting classroom dialogue through developing the Talkwall microblogging tool: considering emerging concepts that bridge theory, practice, and design. NJDL.

[17] Cooper KM, Schinske JN, Tanner KD (2021). Reconsidering the share of a Think-Pair-Share: emerging limitations, alternatives, and opportunities for research. CBE Life Sci Educ.

[18] Dallimore EJ, Hertenstein JH, Platt MB (2013). Impact of cold-calling on student voluntary participation. J Manag Educ.

[19] Seery MK, Agustian HY, Christiansen FV, Gammelgaard B, Malm RH (2024). Incorporating dialogue in laboratory teaching. Anal Bioanal Chem.

[20] Nallaya S, Gentili S, Weeks S, Baldock K (2024). The validity, reliability, academic integrity and integration of oral assessments in higher education: a systematic review. Issues Educ Res.

[21] Pérez-Higueras JJ, Hidalgo Arroquia JJ, Gancedo-Caravia L (2025). Rubric for peer evaluation of oral presentations: use and perceptions among experienced and non-experienced students. J Dent Educ.

[22] Kasai H, Imafuku R, Shikino K (2025). Learning through oral case presentations and impact on medical students’ clinical training and career development: a mixed-method study. Clin Teach.

[23] Fortune D (2025). It’s time: evidence-based strategies to improve student listening in the college classroom. Coll Teach.

[24] Boudreau JD, Cassell E, Fuks A (2009). Preparing medical students to become attentive listeners. Med Teach.

[25] Edwards-Groves C, Davidson C (2020). Noticing the multidimensionality of active listening in a dialogic classroom. AJLL.

[26] Galikyan I, Admiraal W (2019). Students’ engagement in asynchronous online discussion: the relationship between cognitive presence, learner prominence, and academic performance. Internet High Educ.

[27] Garrison DR, Anderson T, Archer W (1999). Critical inquiry in a text-based environment: computer conferencing in higher education. Internet High Educ.

[28] Lahza HF, Demartini G, Noroozi O, Gašević D, Sadiq S, Khosravi H (2025). Enhancing peer feedback provision through user interface scaffolding: a comparative examination of scripting and self-monitoring techniques. Comput Educ.

[29] Chi MTH, Menekse M, Resnick LB, Asterhan CSC, Clarke SN (2015). Socializing Intelligence Through Academic Talk and Dialogue.

[30] Mukadam A, Suresh S, Jacobs C (2025). Beyond traditional simulation: an exploratory study on the effectiveness and acceptability of ChatGPT‑4o Advanced Voice Mode for communication skills practice among medical students. Cureus.

[31] Anthamatten A, Holt JE, Pfieffer ML (2025). Developing clinical competence through case presentations with artificial intelligence-driven simulation. J Nurse Pract.

[32] Ding D, Muhyiddin B Yusof A (2025). Investigating the role of AI-powered conversation bots in enhancing L2 speaking skills and reducing speaking anxiety: a mixed methods study. Humanit Soc Sci Commun.

[33] Cooper KM, Downing VR, Brownell SE (2018). The influence of active learning practices on student anxiety in large-enrollment college science classrooms. Int J STEM Educ.

[34] Theobald EJ, Hill MJ, Tran E (2020). Active learning narrows achievement gaps for underrepresented students in undergraduate science, technology, engineering, and math. Proc Natl Acad Sci USA.

